# Supplementary material for: Topological analog signal processing
Source: Nat Commun. 2019 May 3;10:2058. doi: 10.1038/s41467-019-10086-3 (PMC6499819; doi:10.1038/s41467-019-10086-3)
Supplement: Supplementary file 1 — Supplementary Information [file 41467_2019_10086_MOESM1_ESM.pdf]

Supplementary Information for the paper

Topological Analog signal processing

Farzad Zangeneh-Nejad and Romain Fleury*

Laboratory of Wave Engineering, EPFL, 1015 Lausanne, Switzerland

**To whom correspondence should be addressed. Email: romain.fleury@epfl.ch*

Supplementary Note 1: Electromagnetic-based topological equation solver

This section describes the extension of the proposed acoustic equation solver to its electromagnetic counterpart. Consider a SSH-like array of silicon rods placed inside a metallic rectangular waveguide whose width and height are $20\ cm$ (Supplementary Figure 1). Through a topological phase transition, a topological edge mode appears at the interface between the two crystals. Upon its excitation, this topological edge mode creates a resonance in the transmission of the waveguide (Supplementary Figure 1a (middle inset)) at the frequency of $f_0 = 1.279\ GHz$. The resonance line shape can be well estimated with the following transfer function for $A = 2.8 \times 10^5$ and $Q = 1.78 \times 10^{-6} f_0$

$$H(f) = \frac{A}{j(f - f_0) + \frac{f_0}{2Q}} \tag{S1}$$

which corresponds to the following ODE

$$\begin{aligned} &f'(\mathrm{t}) + \frac{2\pi f_0}{2Q} f(\mathrm{t}) = 2\pi A g(\mathrm{t}) \\ &f(0) = 0 \end{aligned} \tag{S2}$$

Now consider a Gaussian type ($\sigma = 1\ MHz$) input signal $g(t)$ modulated at the carrier frequency f_0 (left panel). Applying the transfer function $H(f)$ (middle panel) to the input signal, one obtains

the corresponding output signal $\tilde{f}(t)$ (right panel), whose envelope is indeed the solution of the differential equation in Eq. S2 (dashed black line). The inset of panel b repeats the same analysis when some disorder is added to the sample by randomly moving the silicon rods. It is observed that, despite the large level of the disorder, neither $H(f)$ nor $\tilde{f}(t)$ has significantly changed, confirming the high stability of the equation solver.

Supplementary Note 2: Finite difference time domain simulation of the proposed equation solver

This section reports on the finite difference time domain (FDTD) simulations of the proposed topological acoustic equation solver. As mentioned, the output signals $\tilde{f}(t)$ in Fig. 2 of the main manuscript were all obtained by applying the corresponding transfer functions $H(f)$ to the input signal $\tilde{g}(t)$, ignoring the time delay between $\tilde{f}(t)$ and $\tilde{g}(t)$. An alternative approach is to obtain $\tilde{f}(t)$ directly from FDTD simulations using Comsol Multiphysics (see Supplementary Figure 2). One notices that the obtained results are identical to those reported in the main text which were obtained from frequency domain simulations.

Supplementary Note 3: Response of the proposed equation solver to a Gaussian input signal

This section examines the behavior of the proposed topological equation solver when a Gaussian input signal modulated at f_0 is considered as the input, instead of the complex pulse chosen in the main text. Supplementary Figure 3 repeats the results of Fig. 2 of the main manuscript for an input signal $\tilde{g}(t)$ with a Gaussian time profile ($\sigma = 60$). The output signal is obtained by applying the transfer function of the system to the input. It is observed that the topological equation solver

works properly with or without position shift disorder, offering a strong stability. As always, the trivial equation solver only works properly in the absence of position shift disorder.

Supplementary Note 4: Measured response of the fabricated equation solver to a Gaussian type input signal

In this section, we experimentally examine the behavior of the fabricated topological equation solver for a Gaussian type input signal. Supplementary Figure 4 repeats the results of Fig. 4 of the main manuscript for a Gaussian type input signal $g(t)$ with $\sigma = 60$. Again, the topological equation solver survives large level of disorder, whereas the performance of the trivial equation solver severely affects when the disorder is added to the system.

Supplementary Note 5: Approaches to control the ODE constant coefficients

Here we discusses different approaches to control the coefficients of the ODE solved by the equation solver. The first and easiest way to do so is to increase the dissipation losses of the system so as to change the quality factor of the topological resonating state. Consider again the SSH array of cylindrical obstacles (Supplementary Figure 5a) used to solve our desired first order differential equation. As already mentioned in the main text, we have neglected the dissipation losses in this configuration. Now we increase the dissipation losses a little bit and plot the resulting transfer function in Supplementary Figure 5b. As expected, the total quality factor of the resonance has been decreased with respect to the lossless case. The new transfer function of the system can indeed be well estimated with $(f) = 1/(j(f - f_0) + 2)$, which corresponds to an ODE of the form $f'(t) + 4\pi f(t) = 2\pi A g(t)$.

The second approach to control the quality factor of the topological resonance is to change the distances between the cylinders. Supplementary Figure 5c and Supplementary Figure 5d represent how the transfer function of the system changes from $H(f) = 1/(j(f - f_0) + 1)$ to $H(f) = 1/(0.5j(f - f_0) + 1)$ when moving from a SSH array with largely detuned array to a less deformed one. The corresponding ODE to the latter case takes the form of $f'(t) + 4\pi f(t) = 4\pi A g(t)$.

The third approach is to increase or decrease the number of unit cells of the SSH array. Obviously, smaller number of unit cells gives rise to a topological resonance with a broader linewidth (or smaller quality factor). This is evident from the results of Supplementary Figures 5e and f, where the transfer function of the system is changed from $H(f) = 1/(j(f - f_0) + 1)$ to $H(f) = 1/(0.125j(f - f_0) + 1)$ as a result of decreasing the number of unit cells from 3 to 2. The ODE corresponding will be of the form $f'(t) + 16\pi f(t) = 16\pi A g(t)$.

We also highlight that, employing a combination of these approaches, allows one to have a wide control over the constant coefficient of the ODE solved by the equation solver.

Supplementary Note 6: Higher order ODE solvers

The scheme described in the main text for solving a second order differential equation is readily extendable to higher order equation solvers. Consider a differential equation of order nth of the form

$$f^{(n)}(t) + B_{n-1} f^{(n-1)}(t) + \ldots + B_1 f'(t) + B_0 f(t) = Ag(t) \tag{S3}$$

Taking Fourier transform of both sides of equation yields the following expression for the transfer function

$$H(f)=\frac{\frac{A}{(2\pi)^n}}{(j(f\text{ - }f_0))^n+\frac{1}{2\pi}B_{n-1}(j(f\text{ - }f_0))^{n-1}+...+\frac{1}{(2\pi)^{n-1}}B_1(j(f\text{ - }f_0))+\frac{1}{(2\pi)^n}B_0} \tag{S4}$$

which can again be decomposed into partial fractions as

$$H(f)=\sum_{i=1}^{n}H_i(f) \tag{S5}$$

where

$$H_i(f)=\frac{A_{\mathrm{i}}}{j(f\text{ - }f_0)+\frac{f_0}{2Q_{\mathrm{i}}}};\ i=1,2,...,n \tag{S6}$$

Where $Q_i=-\frac{f_0}{2P_i}$, in which P_i are the complex poles of the following nth order polynomial

$$x^n+\frac{1}{2\pi}B_{n-1}x^{n-1}+...+(\frac{1}{2\pi})^{n-1}B_1x+(\frac{1}{2\pi})^nB_0=0 \tag{S7}$$

and A_i is of the form

$$A_i=\frac{\frac{A}{(2\pi)^n}(x-P_i)}{x^n+\frac{1}{2\pi}B_{n-1}x^{n-1}+...+(\frac{1}{2\pi})^{n-1}B_1x+(\frac{1}{2\pi})^nB_0}\,|\,x=P_i \tag{S8}$$

Eqs. S5 and 6 suggest a straightforward approach to solve a differential equation of nth order: one has to first realize (first order) differential equation solvers corresponding to the transfer functions, and then add (or subtract) their output signals using rat race couplers as it is accomplished in Supplementary Figure 6.

Supplementary Note 7: Constructing higher order filter transfer functions using topological insulators

In this section, we discuss the possibility of making transfer functions of arbitrary orders making use of topological insulators, by cascading topological boundaries. We start with mentioning the fact that the proposed equation solver in the main text can indeed be pictured as a first order pass-band filter, whose pole is topologically protected by the chiral symmetry of the SSH array. A straightforward approach to achieve higher order filter transfer functions, already discussed in the main text, is to add or subtract the responses of first order filters to or from each other. Here we suggest an alternative route to this goal, based on cascading several SSH arrays with each other. Shown in Supplementary Figure 7a (top) is two cascaded SSH arrays, each of which supports a topological edge mode (resonating at ω_0 with some decay rate of γ for example) at its phase transition boundary. Assuming the coupling coefficient between the two topological edge modes to be ϑ, for example, the transfer function of the overall chain then reads

$$H(\omega) = \frac{1}{-(\omega-\omega_0)^2 + 2j\gamma(\omega-\omega_0) + \vartheta^2 - \gamma^2} \tag{S9}$$

which is nothing but the transfer function of a second order pass-band filter. Supplementary Figure 7a (bottom) represents the transfer function of the coupled SSH chain under investigation, calculated by means of standard tight binding formalism. The obtained transmission coefficient can be fitted with the theoretical relation given in Eq. S9. To assess the robustness of such a topological filter, we add some disorder to the coupling coefficients between the resonators, and plot in Supplementary Figure 7b the evolution of the (averaged) transfer function versus disorder strength. It is apparent that the filter response is affected very minorly by the disorder. To make a comparative case, we couple two trivial resonating defect modes, forming at the boundaries between crystals with opposite on-site potential organizations, as shown in Supplementary Figure

7c. Inset of Supplementary Figure 7d manifests the extreme sensitivity of such a topologically trivial filter to the applied disorder (note that we have used same type and amount of disorder in both trivial and topological cases).

It should be noted that the system under investigation can be treated as a second order equation solver as well. More specifically, the transfer function given in Eq. S9 corresponds to the following second-order ODE

$$\frac{d^2 f(t)}{dt^2} + 2\gamma \frac{df(t)}{dt} + (\vartheta^2 - \gamma^2) f(t) = g(t) \tag{S10}$$

To demonstrate such functioning, we consider in Supplementary Figure 8b an input signal with a Gaussian temporal evolution to be applied to the array, and calculate the corresponding output (Supplementary Figure 8d) by applying the transfer function of the array (Supplementary Figure 8c) to the input signal. Comparing the obtained output signal with the solution of Eq. S10 (its envelope) proves the expected functioning of the system. Notice that such an equation solver is protected to perturbation of the hopping amplitudes as already demonstrated in Supplementary Figure 7b.

Supplementary Note 8: Possibility of spatial analog computing using topological insulators

In this section we provide a proposal for performing spatial analog computing using topological insulators. Consider a SSH array of plasmonic graphene ribbons, placed on top of a silicon dioxide substrate (Supplementary Figure 9a). Such an array of resonators supports a topological edge mode whose profile is represented in Supplementary Figure 9b. Suppose now that an incident field E_i impinges the structure at the resonance frequency of the topological edge mode. Supplementary Figure 9c shows the reflection coefficient of the structure versus the incident angle θ, calculated via full-wave numerical simulations. It is apparent that, at the incident angle of around $\theta = 8°$, the reflection coefficient vanishes. Such a zero in the reflection coefficient of the structure (which is

due to the impedance matching between the topological edge mode and the incident field), can be leveraged to differentiate the impinging signal as described in [1-3]. To verify such a functionality, we consider a Gaussian beam to be incident on the structure (Supplementary Figure 9d), and analytically calculate the corresponding reflected field (Supplementary Figure 9e). It is obvious that the reflected field possesses a Gaussian derivative spatial distribution, approximating well the spatial differentiation of the incident field.

Supplementary Note 9: Comparison between the proposed topological and trivial equation solvers

In this section, we demonstrate that adding disorder to a trivial equation solver severely affects its functioning. To this end, we start with considering a trivial defect mode forming at the interface between two crystals with opposite on-site potential organizations (referred to as A-B and B-A in Supplementary Figure 10). The spectrum of the array is analytically calculated using tight binding equations and represented in Supplementary Figure 10a (bottom panel), showing a resonance peak inside the band gap of the crystal. Supplementary Figure 10b illustrates how the line-shape of this resonance evolves when some disorder (same type and amount as Fig. 3 of the main text) is added to the hopping amplitudes between the resonators. In contrast to the result of Fig. 3 of the main text, we observe that the line-shape is not robust to the added disorder, manifesting the superiority of topological analog signal processing over trivial one. Likewise, we report in Supplementary Figures 8c and d the behavior of the line-shape of the Bragg defect mode versus disorder strength. Comparing the results of these figures with Fig. 3e of the main text, one realizes that the spectral line-shape of the trivial resonance is much more sensitive to the applied disorder than that of the topological one (notice that we have used the same type and amount of disorder in both cases).

Supplementary Figures

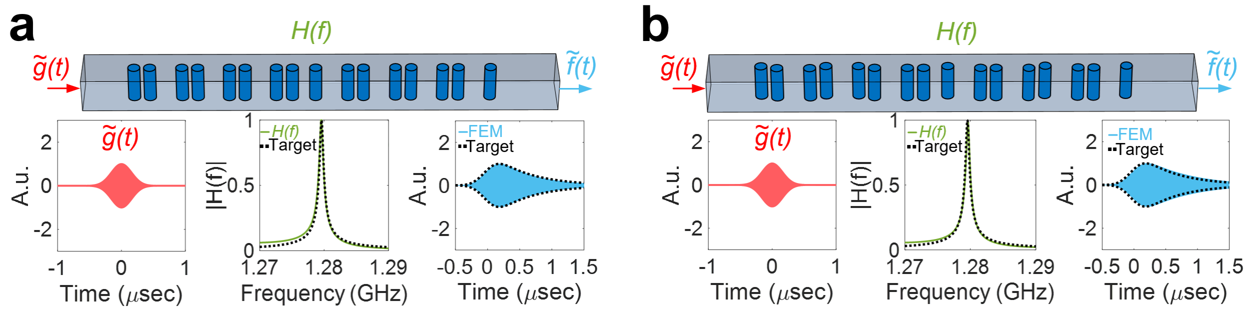

Supplementary Figure 1: Demonstration of a topological microwave equation solver, a, A Gaussian ($\sigma = 1\ MHz$) input signal (left panel) modulated at f_0 is applied to the proposed topological resonator with the transfer function $H(f)$ (middle panel). The envelope of the output signal $\tilde{f}(t)$ (right panel) is the solution of the ODE in Eq. S2. **b,** Same as panel a except that some disorder is added to the system by randomly moving the cylinders. Thanks to its topological properties, the equation solver provides a strong stability against position disorder.

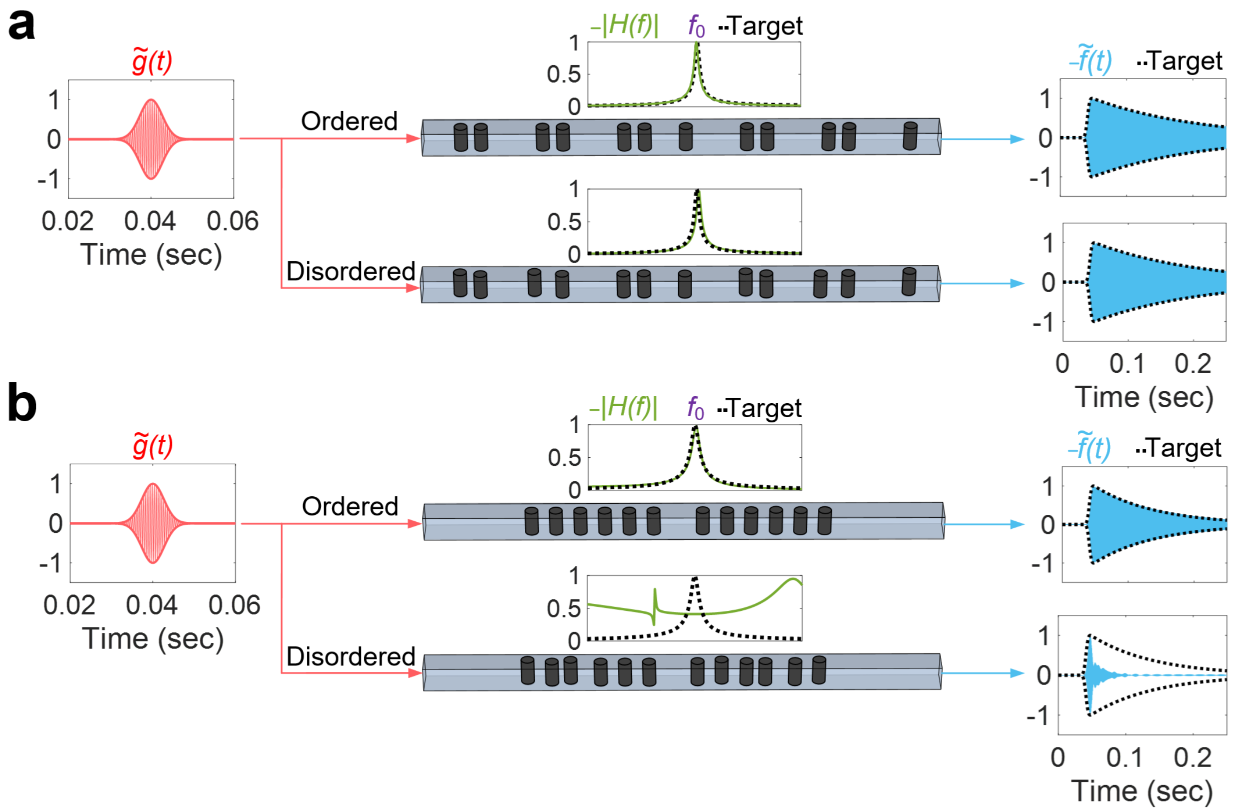

Supplementary Figure 2: Demonstration of the proposed topological equation using direct FDTD simulations. The figure repeats the results of Fig. 2 of the main manuscript, for a Gaussian input signal, except that the output signals are obtained by direct FDTD simulations. **a,** Response of the topological equation solver to a Gaussian input signal. **b,** Response of the trivial equation solver to the same signal.

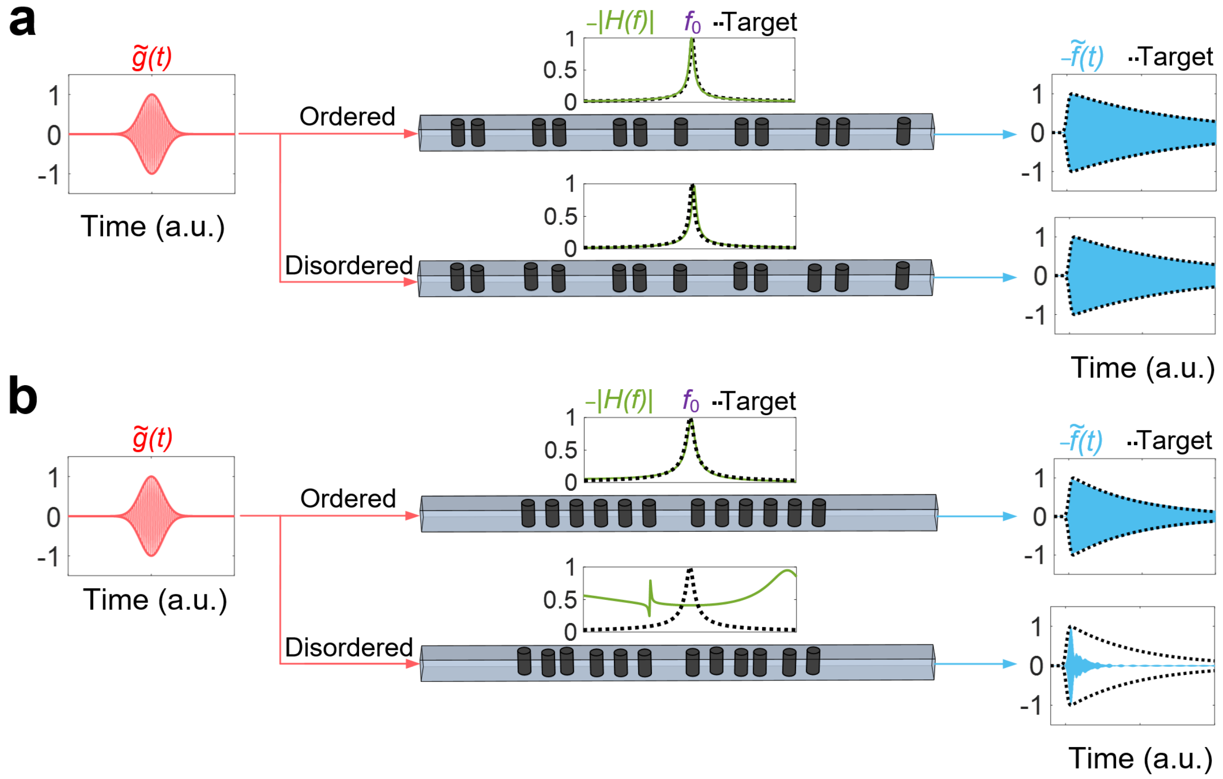

Supplementary Figure 3: Demonstration of the proposed topological equation for a Gaussian type input signal. The figure repeats the results of Fig. 2 of the main manuscript for a Gaussian type input signal with $\sigma = 60$. **a,** The topological equation solver works properly with or without position shift disorder, offering a strong stability. **b,** In contrast, the trivial equation solver only works properly in the absence of disorder.

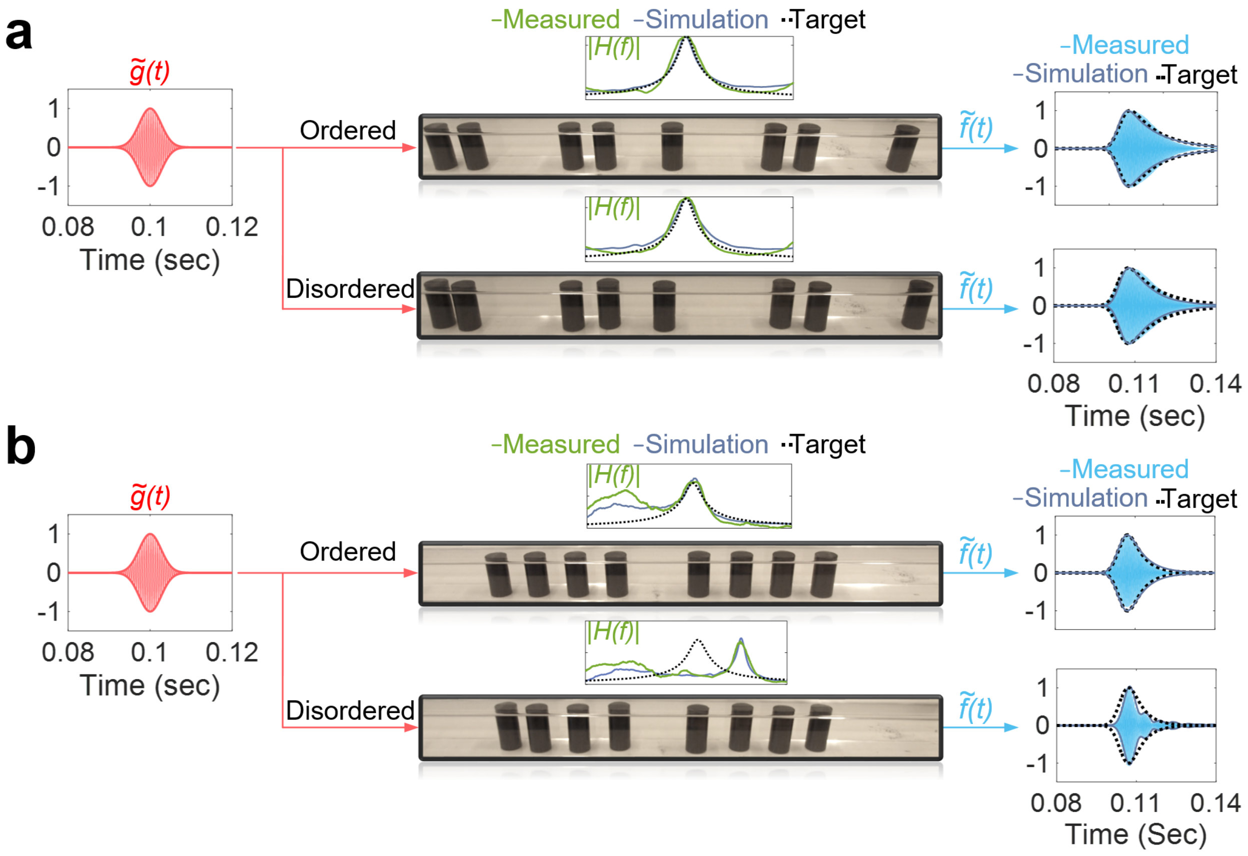

Supplementary Figure 4: Experimental demonstration of the fabricated topological equation solver for a Gaussian type input signal. The figure repeats the results of Fig. 4 of the main manuscript in the case of a Gaussian input signal. **a,** The topological equation solver works properly with or without position shift disorder, offering a strong stability. **b,** In contrast, the trivial equation solver only works properly in the absence of disorder.

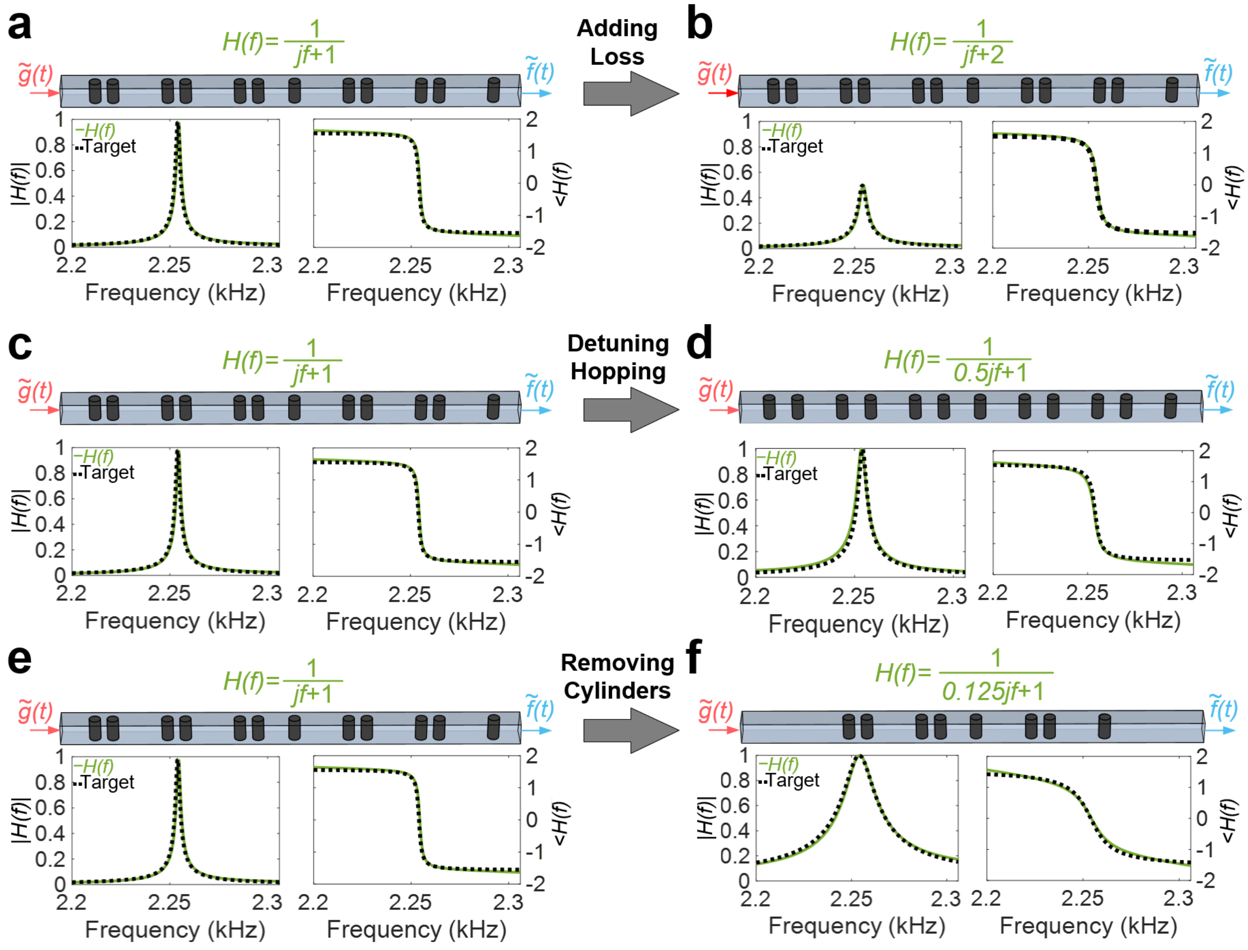

Supplementary Figure 5: Approaches to control the coefficient of the first order ODE solved by the proposed topological equation solver, a,b The transfer function of the equation solver is changed by increasing the dissipation losses of the system. **c,d** The transfer function of the ODE solver is changed by detuning the hopping distances between the cylinders. **e,f** The transfer function of the ODE solver is changed by decreasing the number of unit cells in the SSH array.

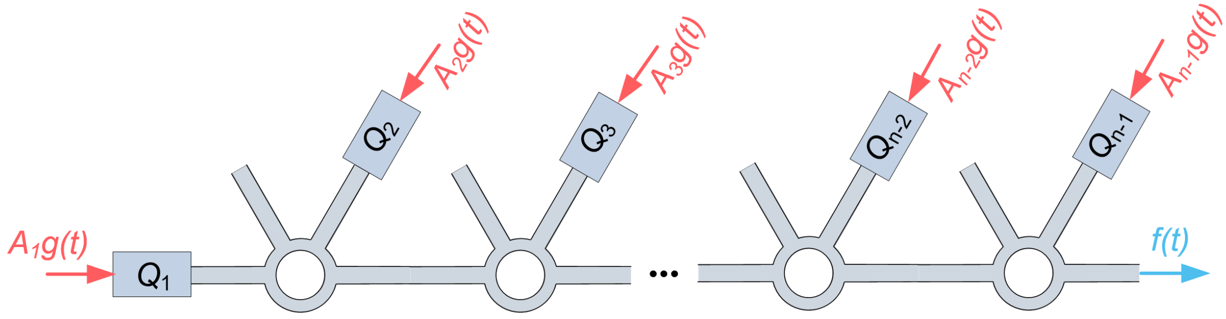

Supplementary Figure 6: Realizing higher order topological ODE solvers: The output signals of n different first order ODE solvers are subtracted using rat race couplers. The overall transfer function of the system $H(f)$ corresponds to the transfer function of the nth order ODE.

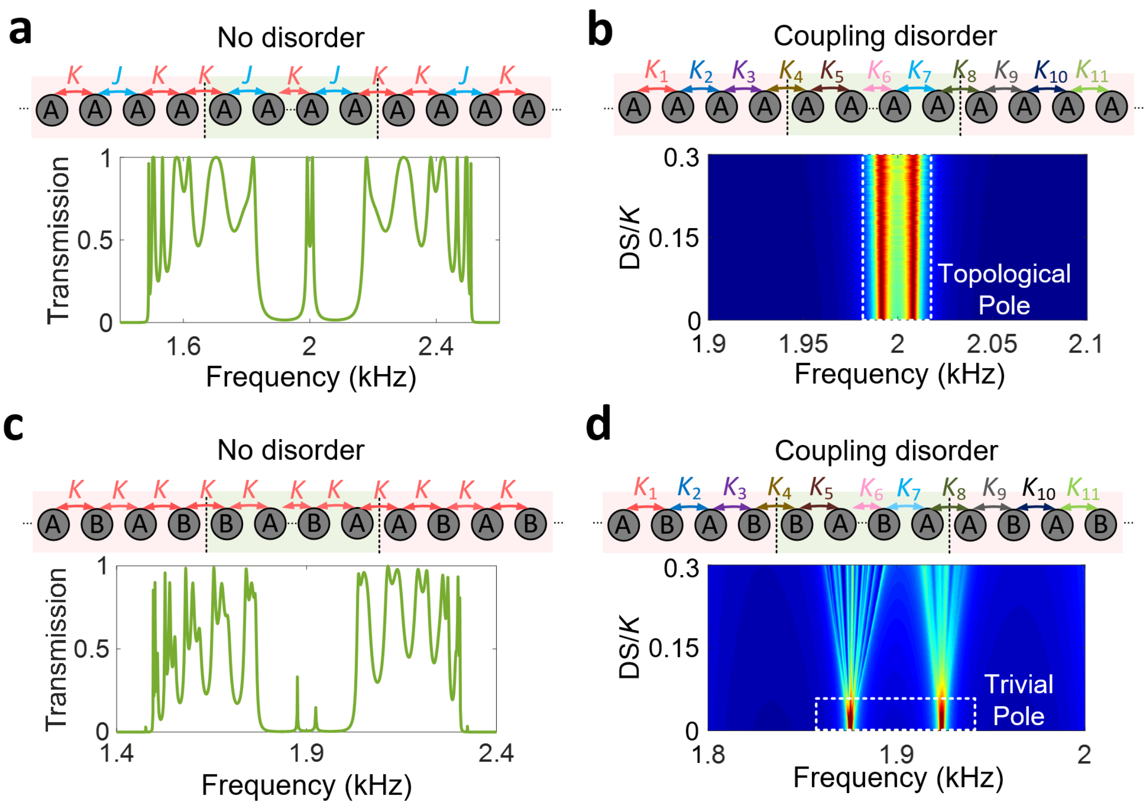

Supplementary Figure 7: Demonstration of a second-order topological pass-band filter, a, Two SSH chain are coupled to each other. The transfer function of the whole chain can be approximated by the theoretical relation given in Eq. S9, corresponding to a second-order pass band filter. **b,** Evolution of the transfer function of the chain, averaged over 20 different realizations of disorder (applied to the hopping amplitudes), versus disorder strength. **c,d,** Same as a,b but for a trivial second order filter.

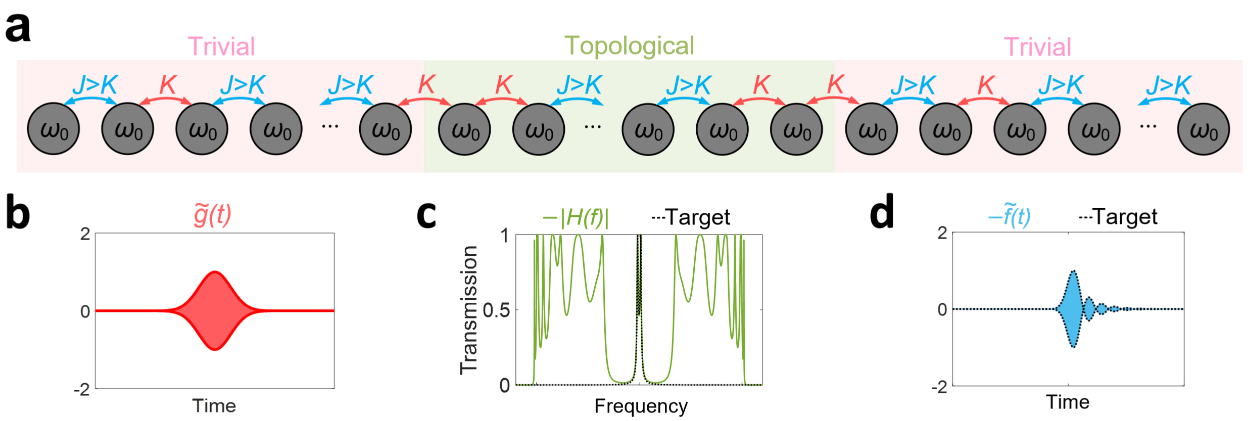

Supplementary Figure 8: Realization of a second order ODE solver by coupling two SSH arrays to each other. a, Two distinct SSH arrays are coupled to each other. **b,** An input signal with a Gaussian distribution is applied to the array. **c,** Transfer function of the whole array. **d,** Output signal of the array, approximating well the solution of the ODE given in Eq. S10.

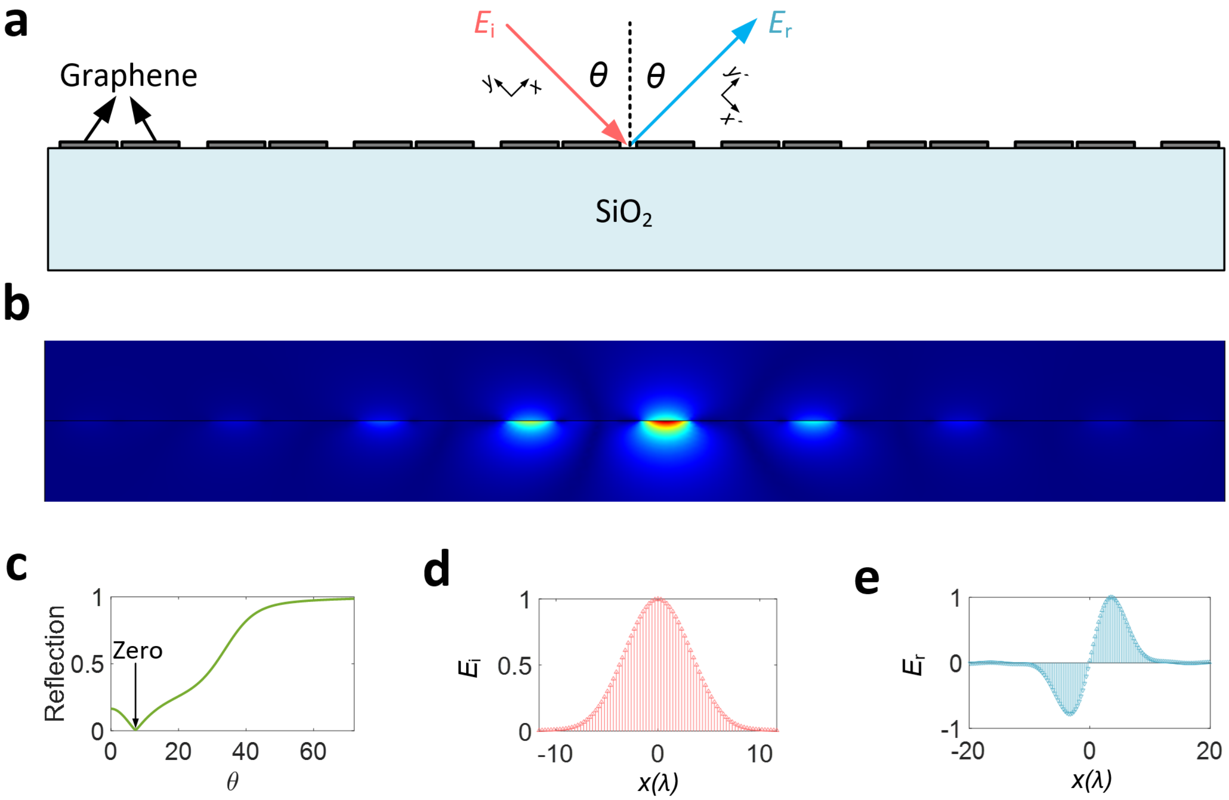

Supplementary Figure 9: Spatial optical computing using topological insulators, a, Consider a SSH array of graphene ribbons placed on top of a silicon dioxide substrate. **b,** Mode profile of the topological edge mode forming at the phase transition boundary of the array, **c,** Reflection coefficient of the structure versus the incident angle. **d,** A Gaussian spatial distribution is assumed for the impinging field. **e,** The reflected field from the structure is analytically calculated, which possesses a Gaussian derivative distribution, approximating well first order differentiation of the incident signal.

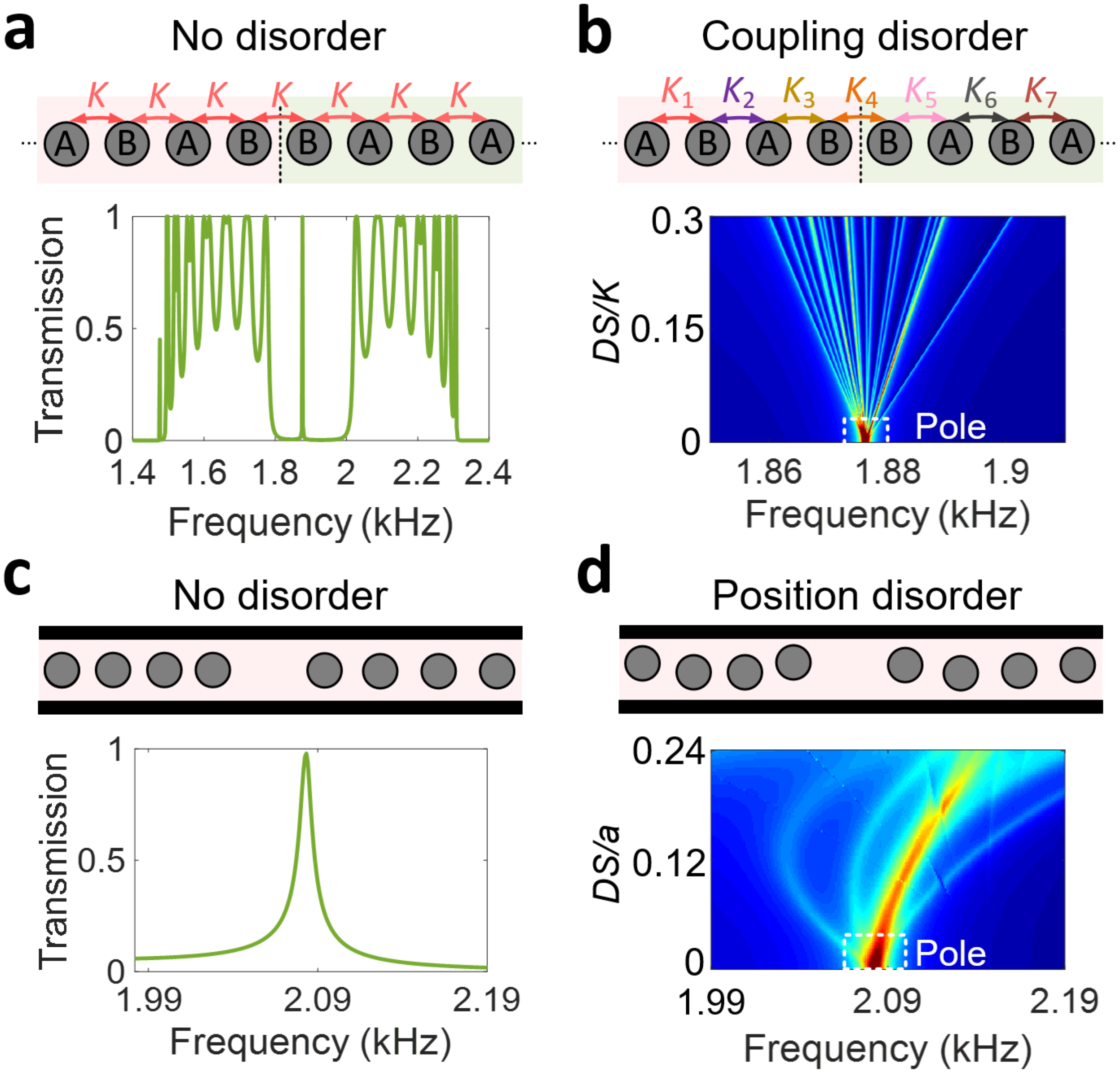

Supplementary Figure 10: Comparison between the topological and trivial equation solvers, a, A trivial defect resonance is achieved by forming an interface between two crystals with opposite on-site potential organizations. **b,** Evolution of the (averaged) spectrum of the trivial resonance versus disorder strength. **c,d,** Same as panel a and b but for a trivial resonance induced by defect tunneling through an acoustic Bragg band gap. Comparing the results of this figure with Fig. 3 of the main text reveals the superiority of topological analog signal processing over trivial one.

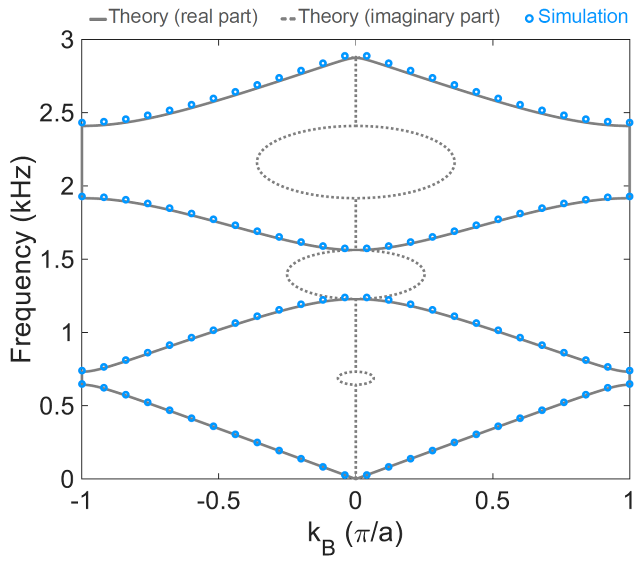

Supplementary Figure 11: **Band structure of the proposed acoustic lattice crystal.** The figures represents the comparison between the crystal band structures obtained from the semi-analytical model based on the transfer-matrix approach (black lines) and from full-wave finite-element simulations.

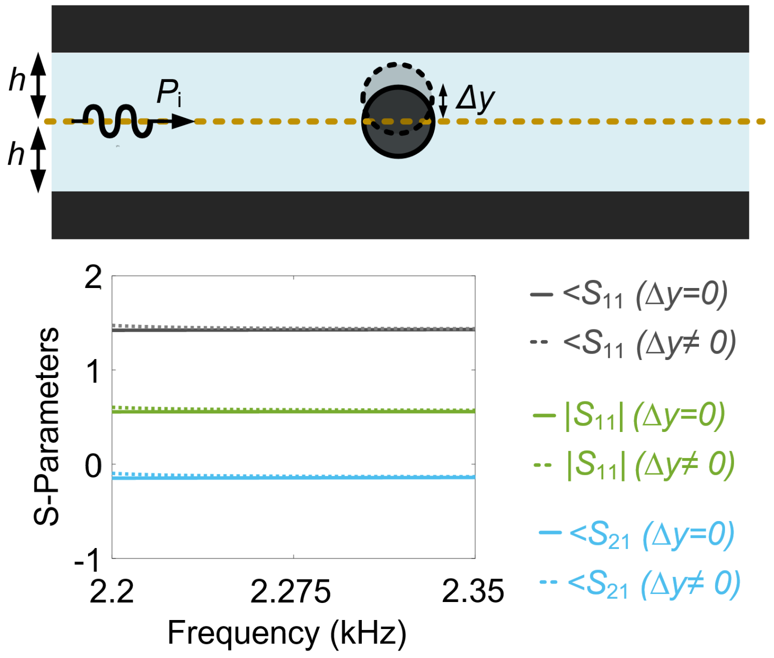

Supplementary Figure 12: **Effect of vertical disorder on the scattering parameters.** Vertical shifts negligibly affect the scattering parameters of the obstacle in the frequency range of interest, effectively preserving the symmetry $M_{\mathrm{cell}}^2 = 1$.

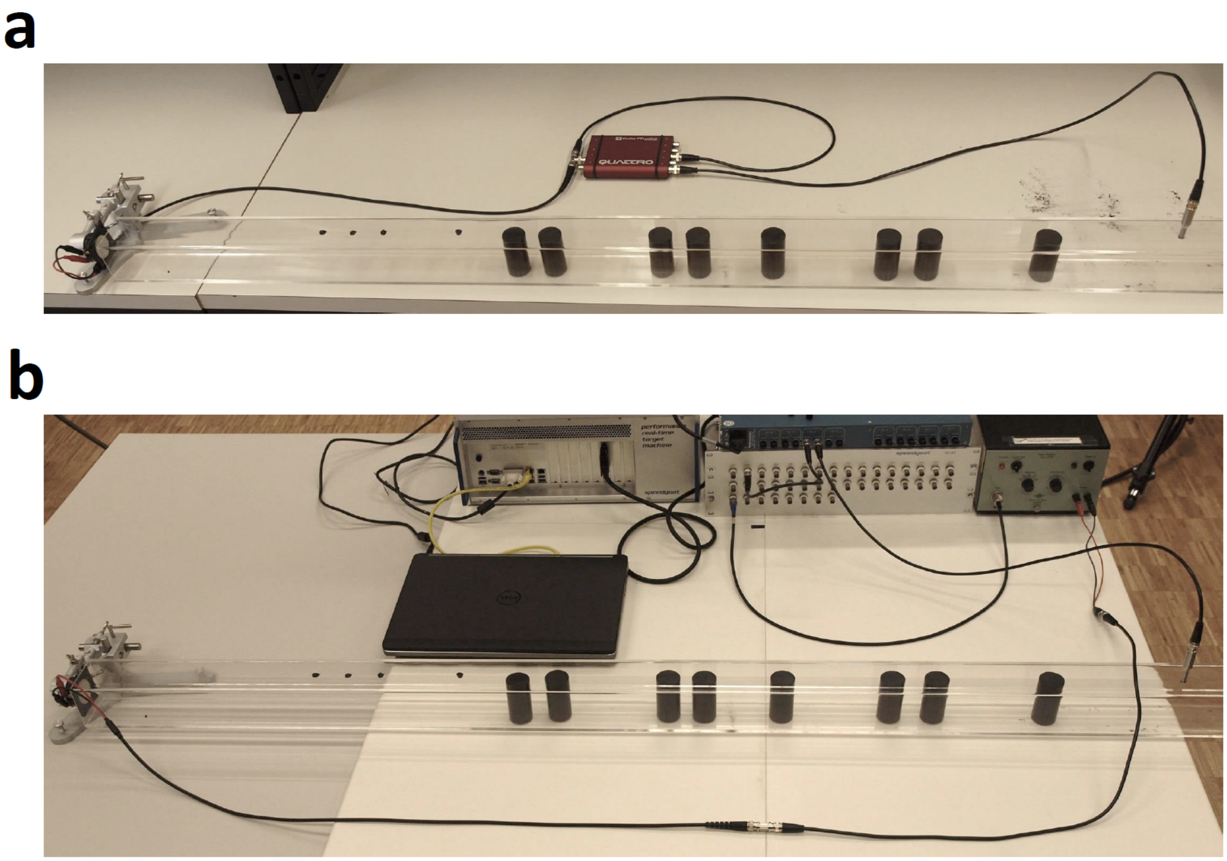

Supplementary Figure 13: Experimental setups a, Experimental setup used to measure the transfer function of the proposed equation solver, **b,** Experimental setup used to create an input signal with an arbitrary time profile and measure the corresponding output signal from the equation solver.

Supplementary References

1. Zangeneh-Nejad, Farzad, Amin Khavasi, and Behzad Rejaei. "Analog optical computing by half-wavelength slabs." Optics Communications **407**, 338-343 (2018).
2. Youssefi, Amir, et al. "Analog computing by Brewster effect." *Optics letters* **41**, 3467-3470 (2016).
3. Zangeneh-Nejad, Farzad, and Romain Fleury. "Performing mathematical operations using high-index acoustic metamaterials." New Journal of Physics **20**, 073001 (2018).
